# Three new species of *Chromis* (Teleostei, Pomacentridae) from mesophotic coral ecosystems of the Philippines

**DOI:** 10.3897/zookeys.835.27528

**Published:** 2019-04-04

**Authors:** B. Gabriela Arango, Hudson T. Pinheiro, Claudia Rocha, Brian D. Greene, Richard L. Pyle, Joshua M. Copus, Bart Shepherd, Luiz A. Rocha

**Affiliations:** 1 Department of Ichthyology, California Academy of Sciences, 55 Music Concourse Dr., San Francisco, CA 94118, USA Department of Ichthyology, California Academy of Sciences San Francisco United States of America; 2 Bernice P. Bishop Museum, Honolulu, HI 96817, USA Bernice P. Bishop Museum Honolulu United States of America; 3 Department of Biology, University of Hawai’i at Mānoa, Hawai’i Institute of Marine Biology, Kãne’ohe, HI 96744, USA University of Hawai’i at Mānoa Kãne’ohe United States of America; 4 Steinhart Aquarium, California Academy of Sciences, 55 Music Concourse Dr., San Francisco, CA 94118, USA Steinhart Aquarium, California Academy of Sciences San Francisco United States of America

**Keywords:** Coral triangle, damselfish, deep reefs, planktivore, rebreather diving, reef fish, taxonomy

## Abstract

﻿Three new species of *Chromis* (Perciformes, Pomacentridae) from the Philippines, collected between 75–150 m depth, are described by a combination of morphological features and their coloration. *Chromisgunting***sp. n.** was found in Batangas and Oriental Mindoro, and differs from its congeners in body depth (2.1–2.2 in SL), and color of adults, light brown, with a silver area on the anterior end and a bilateral black margin along the exterior side of the tail. It is most similar to *C.scotochiloptera*, with a 5.3% genetic divergence in COI. *Chromishangganan***sp. n.** was found around Lubang Island. Body depth (1.9–2.0 in SL) and adult coloration (yellowish with dark black outer margins on dorsal and anal fins) also separate this species from its congeners. It is most similar to *C.pembae*, with a 2.5% genetic divergence. *Chromisbowesi***sp. n.** was found in Batangas, and also differs from its congeners by the combination of body depth (1.5–1.6 in SL), and color of adults (brownish grey in the dorsal side to whitish on the ventral side, with alternating dark and light stripes in the sides of body). It is most similar to *C.earina*, with a 3.6% genetic divergence in COI.

## Introduction

Mesophotic coral ecosystems (MCEs; 30–150 m), also called the coral-reef “twilight zone”, are uncommonly visited and relatively poorly-known deeper extensions of the coral-reef ecosystem (Rocha et al. 2018). Compared to shallow reefs, little is known about MCEs, mostly because they are beyond safe non-technical SCUBA diving depths (Pyle 2000). Submarines, remotely operated vehicles, or technical diving and special equipment (rebreathers and mixed gases) are needed to explore these depths (Pinheiro et al. 2016, Pyle et al. 2016, Baldwin et al. 2018). The rate of new species discovery within MCEs has been high (Pyle 2000), exceeding rates of discovery in any other marine habitat. As we are currently facing a decimation of coral reefs around the world due to anthropogenic impacts (Bellwood et al. 2004, Hughes et al. 2017), it is imperative that we study MCEs in an effort to understand their biodiversity and the threats they face.

The Pomacentridae (damselfishes and anemonefishes) is one of the largest families of reef fishes, with over 330 described species (Michael 2008) and varied morphological characters. The genus *Chromis* Cuvier, 1814, is composed of predominantly planktivorous species, and several studies have shown that the relative abundance of this group increases with depth (Thresher and Colin 1986, Bejarano et al. 2014, Pinheiro et al. 2016, Coleman et al. 2018). *Chromis* is the largest damselfish genus, with 103 described species, at least ten of which are found only on MCEs, below 60m (Allen and Erdmann 2012). The aim of this paper is to describe three new species of *Chromis* discovered during three expeditions conducted between 2013 and 2015 in the Philippines.

## Materials and methods

Specimens were collected during expeditions to Batangas, Lubang, Puerto Galera, and Verde Island, Philippines, organized by the California Academy of Sciences, the Hawaii Institute of Marine Biology, and the Bishop Museum from 2013 to 2015. The research team used technical diving to access reefs and rocky structures at depths between 60–150 m. All species were collected using hand nets or a Hawaiian sling. The specimens were deposited in the fish collection of the Philippines National Museum of Natural History (**PNM**), the California Academy of Sciences (**CAS**), the Bernice Pauahi Bishop Museum (**BPBM**), and the United States National Museum (**USNM**). Comparative material was obtained from the California Academy of Sciences (CAS) ichthyology collection.

Counts were made using a stereo microscope, and morphological characters were measured with a digital caliper, to the nearest 0.01 mm and rounded to one decimal place, following Allen and Randall (2004) and Pyle et al. (2008). Rudimentary (spiniform) caudal fin-rays are those situated anteriorly to procurrent caudal-fin rays, only visible in X-radiographs. ﻿Vertebral counts include the first vertebra fused to the skull, and the last vertebra fused to the hypural plate. Measurements presented in the text are proportions of standard length (SL). Counts, measurements, and proportions inside parenthesis represent ranges for paratypes, when different from the holotype. Values separated by a pipe character (“|”) represent left|right sides of specimens. Molecular analysis and PCR amplification of the standard barcode fragment of the mitochondrial cytochrome c oxidase subunit I gene (COI) was performed following protocols established by the Center for Comparative Genomics laboratory (Sellas 2014) and compared with more than 60 other species of *Chromis* available from GenBank and the tissue collection at the California Academy of Sciences. Primers used were BOLFishF1/BOLFishR1 for every sample except for CAS 242278, which used FishF2/FishR2, following Weigt et al. (2012). Alignments of DNA sequences were done using the program Geneious 9.1.5 (Kearse et al. 2012). Genetic distances were calculated using Tamura-Nei model (Tamura and Nei 1993). New sequences were deposited in GenBank with the following accession numbers: *Chromisgunting* sp. n. (MH170474), *Chromishangganan* sp. n. (MH170475 and MH170476), and *Chromisbowesi* sp. n. (MH170477, MH170478 and MH170479). Additional new sequences include: holotype of *Chromisearina* Pyle, Earl & Greene, 2008 (MH170472), *Chromispembae* Smith, 1960 (MK049176), and *Chromisscotochiloptera* Fowler, 1918 (MK059781). We also used the following sequences from GenBank for comparisons: *Chromisanalis* Cuvier, 1830 (JF493172.1), *Chromiscinerascens* Cuvier, 1830 (KU944441.1), *Chromisdegruyi* Pyle, Earle & Greene, 2008 (EU358588.1), and *Chromiswoodsi* Bruner & Arnam, 1979 (HM421816.1).

## Results

### Chromis gunting sp. n.

Taxon classificationAnimaliaPerciformesPomacentridae

http://zoobank.org/9BE50EAC-224B-4E20-B40A-19F6BD747EC9

**Figure 1.**
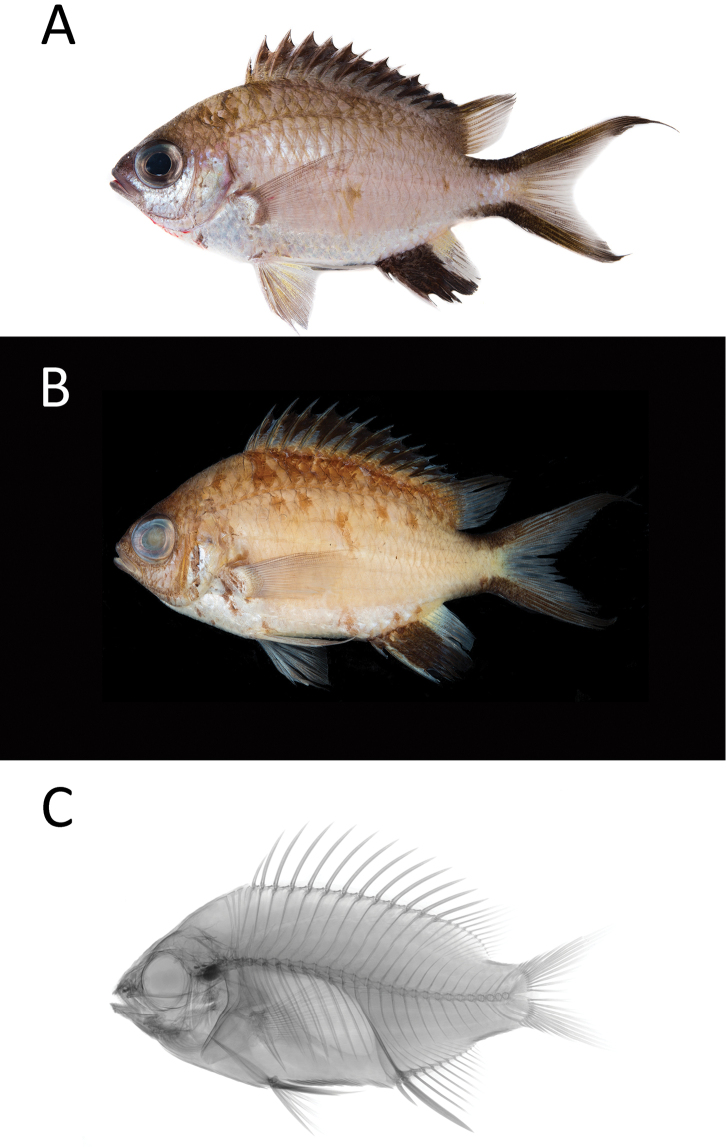
*Chromisgunting* sp. n. PNM 15357 **a** holotype shortly after death, 67.69 mm SL, photograph LA Rocha **b** preserved holotype, photograph JD Fong **c** radiograph of holotype by JD Fong.

Figure 1a–c , Table 1

#### Type material.

**Holotype**: PNM 15357 (field code: LAR 1762). 67.7 mm SL, GenBank MH170474, Layaglayag point, Batangas, Philippines. 13°41'22.74"N, 120°50'12.46"E, 100 m, BD Greene and RL Pyle, 6 December 2013 (Figure 1a–c). **Paratype**: CAS 242328 (field code: HTP 509). 77.4 mm SL, Puerto Galera, Oriental Mindoro, Philippines. 13°31'17.68"N, 120°59'41.78"E, 100 m, LA Rocha, HT Pinheiro, B Shepherd, E Jessup and BD Greene, 9 April 2015.

#### Diagnosis.

The following combination of characters distinguishes *Chromisgunting* sp. n. from all of its congeners: dorsal-fin rays XIII,11; anal-fin rays II,11–12; pectoral-fin rays 16–17; procurrent caudal-fin rays 3; tubed lateral-line scales 14–16; gill rakers 4–5+14–16 (total 19–20); body depth 2.1–2.2 in SL; color of adults when fresh is light brown, with a silver area on the anterior end and a bilateral black margin along the exterior side of the tail.

#### Description.

Dorsal-fin rays XIII, 11 (XIII, 11); anal-fin rays II,11 (II,12); all dorsal and anal-fin rays branched, the last to base; pectoral-fin rays 16 (17), the upper and lowermost unbranched; pelvic-fin rays I,5; principal caudal-fin rays 7+6=13 (6+6=12); upper and lower procurrent caudal-fin rays 3; upper and lower rudimentary (spiniform) caudal-fin rays 2 and 2 respectively (3 and 3); tubed lateral-line scales 16|15 (14|damaged); posterior midlateral scales with a pore 7|7 (5|6); scales above dorsal fin to origin of dorsal fin 3|3; scales below lateral line to origin of anal fin 8|8 (7|7); circumpeduncular scales 12 (11); gill rakers 5+14=19 (4+16=20); ﻿vertebrae 25 (10 precaudal + 15 caudal).

Body depth 2.1 (2.2) in SL, and width 2.6 (2.4) in body depth; head length 3.2 (3.1) in SL; dorsal profile of head with slight convexity anterior to eye, straight dorsal to eye, and slight convexity on nape; snout length 8.0 (6.1) in head length; orbit diameter 2.6 (2.3) in head length; interorbital width 2.7 (2.4) in head length; caudal-peduncle depth 2.1 (2.4) in head length; caudal-peduncle length 2.4 (2.6) in head length.

Mouth terminal, oblique, upper jaw angle of about 35°; maxilla posterior edge vertical at anterior edge of pupil, upper jaw length 2.6 (3.1) in head length; teeth multi-serial, outer row of conical teeth in each jaw, largest anteriorly; narrow band of villiform teeth lingual to outer row, in 2–3 irregular rows anteriorly, narrowing to a single row on side of jaws; tongue triangular with rounded tip; gill rakers long and slender, longest on lower limb near angle almost two-thirds length of longest gill filaments; nostril without fleshy rim, located at level of middle of pupil.

Opercle ending posteriorly in flat spine, tip relatively obtuse and obscured by large scale; preopercle margin smooth, posterior margin extending dorsally to level of upper edge of pupil; suborbital with free lower margin extending nearly to a vertical at posterior edge of pupil. Scales finely ctenoid; anterior lateral line ending beneath rear portion of spinous dorsal fin (between 12^th^ and 13^th^ dorsal-fin spines); head scaled except lips; narrow scaly sheath at base of dorsal and anal fins, progressively wider on soft portion; column of scales on each membrane of dorsal fin, narrowing distally, those on spinous portion of dorsal progressively longer, reaching about half the distance to spine tips on posterior membranes; scales on anal-fin membrane in one column, progressively smaller distally; small scales on caudal fin extending slightly more than three-fourths distance to posterior margin; small scales on basal one-fifth of pectoral fins; median scaly process extending posteriorly from between base of pelvic fins, its length about half that of pelvic spine; axillary scale above base of pelvic spine slightly more or less than one-third length of spine.

#### Color.

Fresh adult specimens (Figure 1a) brown dorsally, changing gradually to pinkish on center of body, to silver ventrally. Black margin along spinous dorsal fin, and on dorsal and ventral margins of caudal fin and peduncle. Soft dorsal fin translucent with dark rays. Anterior two-thirds of anal fin black, posterior with yellowish base and translucent bottom half with dark rays. Pectoral fin base gray, pectoral fin translucent with dark margins on rays. Pelvic fins light brown, almost translucent, with yellow membranes on basal half and gray rays. Color in alcohol similar to live specimen, but body overall browner, and shiny silver portions of anterior ventral portion replaced with whitish silver color.

#### Etymology.

The name *Chromisgunting* sp. n. means scissors in Tagalog, in reference to the bilateral outermost black margins of fish’s caudal fin that gives it the appearance of scissors. To be treated as a noun in apposition.

#### Distribution and habitat.

*Chromisgunting* sp. n. is only known from the Verde Island Passage, in Puerto Galera and Batangas. The species was recorded on MCEs at depths of 90–130 m.

**Table 1.** Percent measurements (%SL) of *Chromisgunting* sp. n., *C.hangganan* sp. n., and *C.bowesi* sp. n. Counts and measurements for the holotype are presented followed by ranges for paratypes (in parentheses). Values separated by a pipe “|” are left|right or upper|lower. Values that do not overlap between species of *Chromis* are in bold.

	* Chromis gunting *		* Chromis hangganan *		* Chromis bowesi *				
	Holotype PNM 15357	Paratype CAS 242328	Holotype PNM 15358	Paratype CAS 243205	Holotype PNM 15359	Paratype CAS 242278	Paratype CAS 242324	Paratype BPBM 41350	Paratype USNM 440406
Standard length (mm)	67.7	77.4	57.8	47.9	82.1	77.5	66.0	77.5	78.3
Body depth	**48.1**	**45.8**	52.5	50.0	**66.0**	**64.0**	**60.8**	**67.5**	**65.5**
Body width	**18.6**	**19.2**	17.7	16.3	**18.6**	**20.1**	**18.1**	**20.1**	**20.5**
Head length	31.7	32.4	33.1	31.6	30.4	36.7	33.2	30.7	33.2
Snout length	4.0	5.3	6.1	4.4	4.0	5.6	6.5	3.9	4.6
Orbit diameter	12.3	14.3	11.8	11.8	11.9	12.6	12.5	12.9	13.3
Interorbital width	11.8	13.6	11.4	9.7	12.6	12.0	12.0	13.0	13.2
Caudal-ped. depth	15.4	13.4	14.7	15.2	16.3	16.7	15.1	16.1	15.6
Caudal-ped. length	13.5	12.5	13.4	12.7	**10.6**	**9.6**	**8.6**	**10.6**	**8.6**
Upper jaw length	12.1	10.4	10.2	9.5	9.8	10.6	10.3	9.6	9.3
Predorsal length	43.4	45.5	47.4	46.2	44.5	46.0	43.4	46.0	47.1
Spinous dorsal-fin base	47.5	43.3	44.2	43.5	49.6	44.7	46.1	51.3	50.7
Soft dorsal-fin base	13.7	13.2	**16.0**	**16.0**	**18.4**	**20.2**	**17.4**	**18.0**	**17.5**
1^st^ dorsal spine	8.7	9.1	6.9	7.8	8.2	8.4	9.4	7.8	6.6
2^nd^ dorsal spine	14.5	14.0	12.8	14.1	14.8	13.7	13.0	14.6	13.0
3^rd^ dorsal spine	17.3	16.4	15.7	16.0	18.4	17.4	16.4	17.8	15.9
4^th^ dorsal spine	18.2	18.9	19.6	17.8	18.6	18.9	16.9	19.8	18.1
5^th^ dorsal spine	22.3	20.2	17.0	18.3	18.8	20.6	19.4	20.3	17.7
6^th^ dorsal spine	17.3	17.8	**17.0**	**17.1**	17.7	21.8	18.6	19.9	18.2
Last dorsal spine	14.8	11.7	13.1	11.7	12.7	17.5	14.0	13.9	14.6
Longest dorsal ray	23.4	21.7	**17.2**	**18.0**	23.7	27.6	25.7	24.3	24.6
Preanal length	71.5	71.0	77.3	78.7	71.0	76.1	77.4	71.2	73.5
1^st^ anal spine	7.0	7.6	7.4	8.0	7.2	8.7	7.3	7.1	6.5
2^nd^ anal spine	20.6	19.1	21.5	22.2	23.9	26.0	21.7	24.1	21.7
Longest anal ray	21.3	19.4	19.8	21.2	22.0	21.8	23.3	20.0	22.0
Anal-fin base	21.3	19.5	20.8	21.8	**26.6**	**25.9**	**26.04**	**28.0**	**26.1**
Caudal fin length	41.2	32.5	35.0	broken	38.7	44.1	38.3	38.2	38.6
Caudal concavity	21.4	14.3	16.4	broken	18.8	23.6	20.8	19.3	20.2
Longest pectoral ray	28.9	30.8	28.7	31.9	34.0	40.0	38.9	37.7	36.9
Prepelvic length	44.3	42.7	48.6	40.4	42.6	52.0	51.1	44.1	46.0
Pelvic-spine length	**18.5**	**17.9**	18.9	19.7	19.5	20.5	19.9	18.8	18.9
1^st^ pelvic soft ray	26.8	29.3	25.9	21.5	26.9	34.6	34.1	23.7	28.3

### Chromis hangganan sp. n.

Taxon classificationAnimaliaPerciformesPomacentridae

http://zoobank.org/CCF56742-B8B7-410E-B15B-8CD7CBD5CAD0

**Figure 2.**
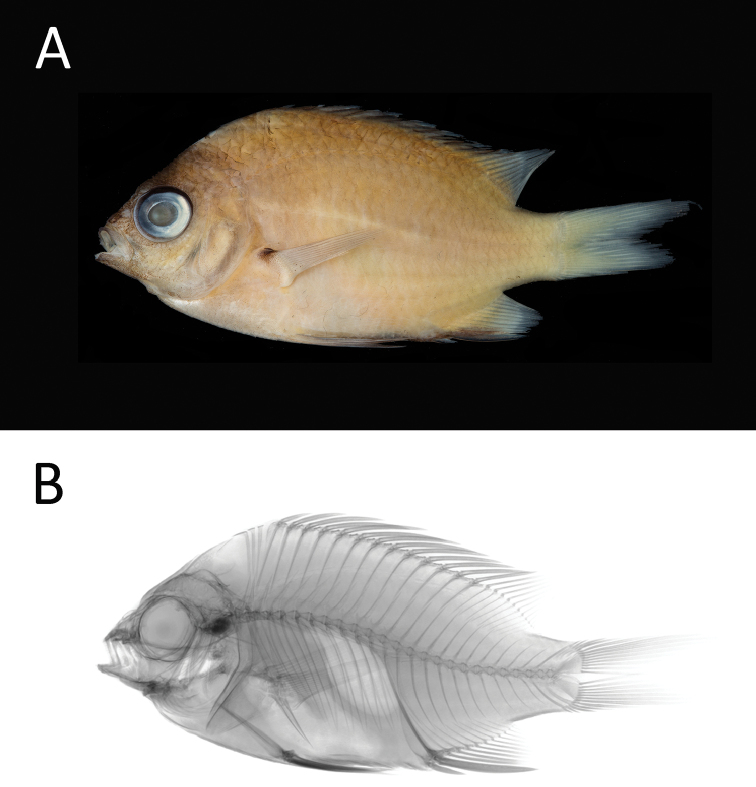
*Chromishangganan* sp. n. PNM 15358 **a** holotype preserved in alcohol, 57.75 mm SL**b** radiograph (photographs JD Fong).

Figure 2a, b , Table 1

#### Type material.

**Holotype**: PNM 15358 (field code: HTP 352). 57.8 mm SL, GenBank MH170475, Lubang, Philippines. 13°45'44.71"N, 120°07'38.31"E, 130 m, RL Pyle and BD Greene, 16 May 2014 (Figure 2a, b). **Paratype**: CAS 243205 (field code: HTP 355). 47.9 mm SL, GenBank MH170476, Lubang, Philippines. 13°45'44.71"N, 120°07'38.31"E, 130 m, RL Pyle and BD Greene, 16 May 2014.

#### Diagnosis.

The following combination of characters distinguishes *Chromishangganan* sp. n. from all of its congeners: dorsal-fin rays XIII, 10–12; anal-fin rays II,11–12; pectoral-fin rays 18; procurrent caudal-fin rays 3; tubed lateral-line scales 16; gill rakers 7+16–19 (total 23–26); body depth 1.9–2.0 in SL; color of adults when fresh yellowish with dark black margins on dorsal and anal fins.

#### Description.

Dorsal-fin rays XIII, 10 (paratype XIII, 12); anal-fin rays II,11 (paratype II,12); all dorsal and anal-fin rays have branched tips; pectoral-fin rays 18, the upper and lowermost unbranched; pelvic-fin rays I,5; principal caudal-fin rays 8+7=15; upper and lower procurrent caudal-fin rays 3; upper and lower rudimentary (spiniform) caudal-fin rays 2 and 1 respectively (paratype 2 and 2); tubed lateral-line scales 16|16; posterior midlateral scales with a pore 9|10 (paratype 10|10); scales above dorsal fin to origin of dorsal fin 3|3 (paratype 2|3); scales below lateral line to origin of anal fin 8|8; circumpeduncular scales 11 (11); gill rakers 7+19=26 (paratype 7+16= 23); ﻿vertebrae 25 (11 precaudal + 14 caudal).

Body depth 1.9 (paratype 2.0) in SL, and width 3.0 (3.1) in body depth; head length 3.0 (3.2) in SL; snout very short, length 5.4 (7.1) in head length; orbit diameter 2.8 (2.7) in head length; interorbital width 2.9 (3.2) in head length; caudal-peduncle depth 2.3 (2.1) in head length; caudal-peduncle length 2.5 (2.5) in head length.

Mouth terminal, small, oblique, upper jaw angle of 35°; maxilla posterior edge beyond anterior edge of eye, upper jaw length 3.3 (3.3) in head length; gill rakers long and slender, longest on lower limb near angle about the length of longest gill filaments; nostril without fleshy rim, located above level of middle of pupil.

Opercle ending posteriorly in internal spine, obscured by scales; preopercle margin smooth, posterior margin extending on the top edge of pupil; suborbital with free lower margin extending nearly to a vertical at posterior edge of pupil. Scales finely ctenoid; anterior lateral line ending beneath the 13^th^ dorsal-fin spine; head scaled except lips, tip of snout, and a narrow zone from orbit to edge of snout containing nostrils; scaly sheath at base of dorsal and anal fins; column of scales on membranes, spines and rays of dorsal and anal fins, progressively smaller distally; small scales on caudal fin extending slightly more than two-thirds distance to posterior margin; small scales on basal one-fifth of pectoral fins; median scaly process extending posteriorly from between base of pelvic fins, its length slightly more than half that of pelvic spine; axillary scale above base of pelvic spine slightly more than one-fifth length of spine.

#### Color.

Images of fresh specimens not available. Our field notes characterize fresh specimens of *Chromishangganan* sp. n. as having a yellowish body color, with light yellow caudal peduncle and caudal fin. Body overall brown, darker dorsally. Black outer margins of the spinous dorsal and anal fins, and yellowish dorsal rays. Ventral margin of body and anal fin spines also black. Anal-fin rays yellowish. Anterior (mouth and snout) and dorsal (nape) portions of the head and orbit dark brown. Ventral portion of head light brown. Black dot on dorsal 1/5 of pectoral fin base and axilla.

#### Etymology.

The name *hangganan* means border in Tagalog, in reference to the black margins of the dorsal and anal fins. To be treated as a noun in apposition.

**Distribution and habitat.** Lubang Island, Philippines. Despite dives in several sites of the Verde Island Passage (VIP), Philippines, this species has not been collected outside of Lubang. The Lubang Island environment differs from the other survey sites in the Verde Island Passage by having more exposed and clearer oceanic waters, probably due to its proximity to the South China Sea. The species was recorded on MCEs at depths of 90–130 m.

**Table 2.** COI genetic Tamura-Nei divergence between *C.gunting* sp. n., *C.hangganan* sp. n., *C.bowesi* sp. n., and their closest relatives available from GenBank. Closest divergences are in bold.

	*** C. earina ***	*** C. analis ***	*** C. gunting ***	*** C. cinerascens ***	*** C. degruyi ***	*** C. hangganan ***	*** C. pembae ***	*** C. bowesi ***	*** C. scotochiloptera ***
* Chromis analis *	12.05								
*Chromisgunting* sp. n.	10.67	8.90							
* Chromis cinerascens *	12.74	11.41	5.98						
* Chromis degruyi *	5.59	10.83	10.28	12.32					
*Chromishangganan* sp. n.	10.50	8.90	8.22	9.06	10.59				
* Chromis pembae *	10.50	9.05	9.13	9.06	10.44	**2.55**			
*Chromisbowesi* sp. n.	**3.61**	12.27	11.42	12.86	**4.52**	10.84	10.84		
* Chromis scotochiloptera *	12.22	10.43	**5.33**	**3.44**	11.22	8.74	8.81	11.90	
* Chromis woodsi *	9.98	**7.96**	7.02	9.42	9.64	7.96	**8.74**	**11.08**	**8.42**

### Chromis bowesi sp. n.

Taxon classificationAnimaliaPerciformesPomacentridae

http://zoobank.org/25F8CF1E-1DF8-469A-B5A9-D2D9C20EA863

**Figure 3.**
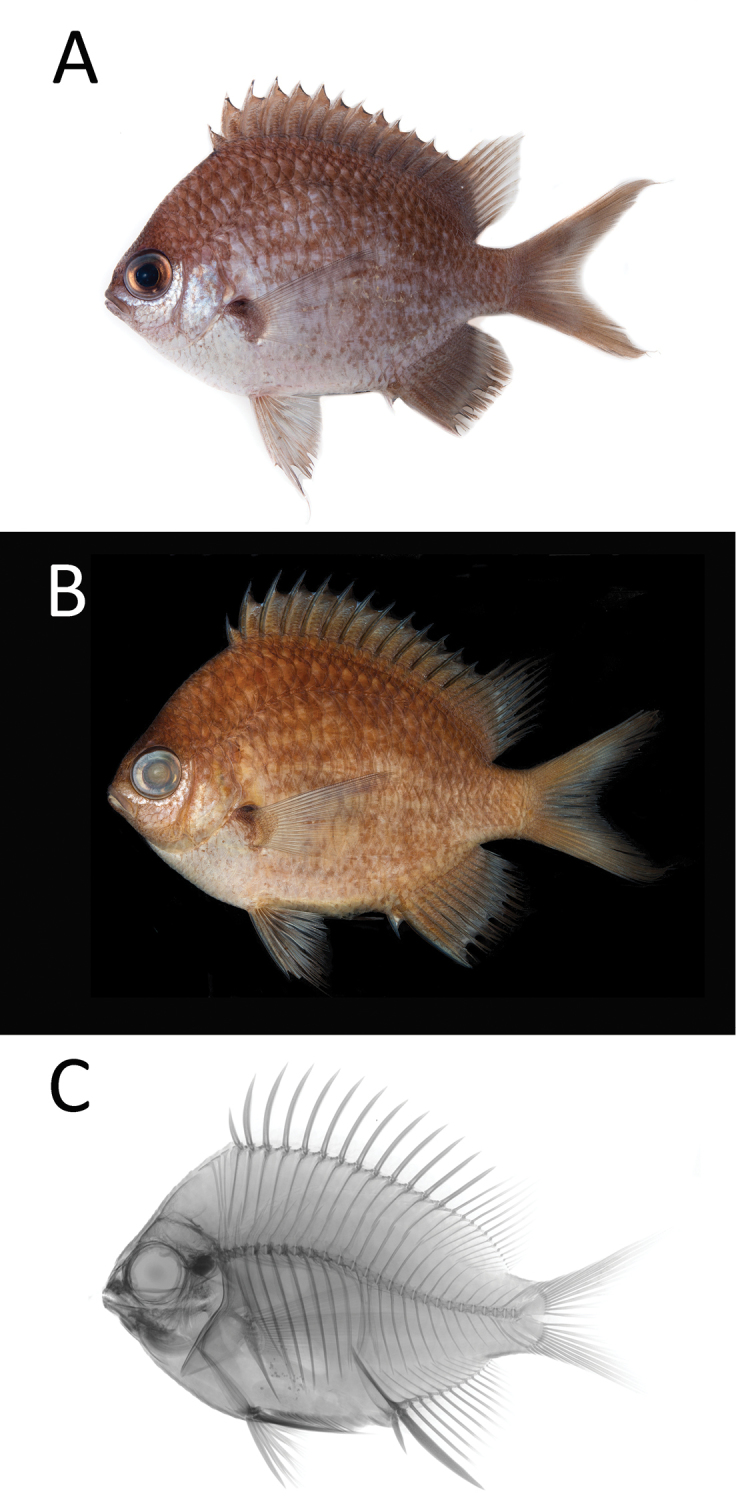
*Chromisbowesi* sp. n. PNM 15359 **a** holotype shortly after death, 82.07 mm SL, photograph LA Rocha **b** preserved specimen, photograph JD Fong **c** radiograph JD Fong.

Figure 3a–c , Table 1

#### Type material.

**Holotype**: PNM 15359 (field code: LAR 1851). 82.1 mm SL, GenBank MH170477, Dive and Trek, Batangas, Philippines. 13°48'3.52"N, 120°54'38.88"E, 120 m, RL Pyle and BD Greene, 12 December 2013 (Figure 3a–c). **Paratypes**: BPBM 41350 (field code: LAR 1852), 77.5 mm SL, GenBank MH170478, Dive and Trek, Batangas, Philippines. 13°48'3.52"N, 120°54'38.88"E, 120 m, RL Pyle and BD Greene, 12 December 2013. USNM 440406 (field code: LAR 1869), 78.3 mm SL, GenBank MH170479, Dive and Treck, Batangas, Philippines. 13°48'3.52"N, 120°54'38.88"E, 120 m, RL Pyle and BD Greene, 14 December 2013. CAS 242324 (field code: HTP 511), 66.0 mm SL, Puerto Galera Bay, Oriental Mindoro, Philippines. 13°31'339"N, 120°960'E, 105 m, LA Rocha, HT Pinheiro, B Shepherd, E Jessup, and BD Greene, 9 April 2015. CAS 242278 (field code: HTP 524), 77.5 mm SL, Verde Island, Batangas, Philippines. 13°31'941"N, 121°06'196"E, 110 m, LA Rocha, HT Pinheiro, E Jessup, and BD Greene, 12 April 2015.

#### Diagnosis.

The following combination of characters distinguishes *Chromisbowesi* sp. n. from all of its congeners: dorsal-fin rays XIII,11–12; anal-fin rays II,11–13; pectoral-fin rays 17–19; procurrent caudal-fin rays 3; tubed lateral-line scales 13–15; gill rakers 6–9+18–19 (total 25–27); body depth 1.5–1.6 in SL; color of adults when fresh brownish grey in the dorsal side to whitish on the ventral side, with alternating dark and light stripes in the sides of body.

#### Description.

Dorsal-fin rays XIII, 12 (two paratypes XIII, 11); anal-fin rays II,12 (one paratype II,11 and two paratypes II,13); all dorsal and anal-fin rays branched, the last to base in some specimens; pectoral-fin rays 18 (one paratype 17 and other 19), the upper and lowermost unbranched; pelvic-fin rays I,5; principal caudal-fin rays 8+7=15 (one paratype 7+6=13 and other 7+7=14); upper and lower procurrent caudal-fin rays 3; upper and lower rudimentary (spiniform) caudal-fin rays 2 and 3 respectively (three paratypes 2 and 2 and one 3 and 2); tubed lateral-line scales 14|14 (other paratypes 13|13, 13|14 and 14|15); posterior midlateral scales with a pore 8 |8 (three paratypes 7|7 and one 7|8); scales above dorsal fin to origin of dorsal fin 3|3 (one paratype 2|2); scales below lateral line to origin of anal fin 8|8; circumpeduncular scales 12 (12); gill rakers from two paratypes 9+18=27 and 6+19=25; ﻿vertebrae 26 (11 precaudal + 15 caudal).

Body depth 1.5 (paratypes 1.5–1.6) in SL, and width 3.5 (3.2–3.4) in body depth; head length 3.3 (2.7–3.3) in SL; dorsal profile of head with slight convexity anterior to eye, slight concavity dorsal to eye, and slight convexity on nape; snout very short, length 7.5 (5.1–7.9) in head length; orbit diameter 2.6 (2.4–2.9) in head length; interorbital width 2.4 (2.4–3.1) in head length; caudal-peduncle depth 1.9 (1.9–2.2) in head length; caudal-peduncle length 2.9 (2.9–3.9) in head length.

Mouth terminal, small, oblique, upper jaw forming an angle of 50°; maxilla posterior edge beyond anterior edge of orbit, upper jaw length 3.1 (3.2–3.6) in head length; teeth multi-serial, outer row of conical teeth in each jaw, largest anteriorly; narrow band of villiform teeth lingual to outer row, in two irregular rows anteriorly, narrowing to a single row on side of jaws; tongue oblong with rounded tip; gill rakers long and slender, longest on lower limb near angle about the same length of longest gill filaments; nostril with fleshy elevated rim, located above level of middle of pupil.

Opercle ending posteriorly in flat spine, tip relatively obtuse and obscured by large scale; preopercle margin smooth, posterior margin extending dorsally to level of upper edge of pupil; suborbital with free lower margin extending nearly to a vertical at posterior edge of pupil. Ctenoid scales; anterior lateral line ending beneath rear portion of spinous dorsal fin (between 10^th^ and 11^th^ dorsal-fin spines); head scaled except lips, tip of snout, and a narrow zone from orbit to edge of snout containing nostrils; narrow scaly sheath at base of dorsal and anal fins, about two-thirds pupil diameter at base of middle of spinous portion of dorsal fin, progressively narrower on soft portion; two columns of scales on each membrane of dorsal fin, narrowing distally, those on spinous portion of dorsal progressively longer, reaching about two-thirds distance to spine tips on posterior membranes; scales on anal-fin membrane in two columns, progressively smaller distally; small scales on caudal fin extending slightly more than one-third distance to posterior margin; small scales on basal one-fifth of pectoral fins; median scaly process extending posteriorly from between base of pelvic fins, its length about half that of pelvic-fin spine; axillary scale above base of pelvic-fin spine large, slightly more than two-thirds length of spine.

#### Color.

Within its natural habitat, *Chromisbowesi* sp. n. has purplish blue body, with distinctive vertical faint light blue bar just behind pectoral fin. Spinous dorsal and pelvic fins light blue to white; soft dorsal translucent. Specimens photographed shortly after death (Figure 3a) are dark grayish brown dorsally, fading to light silvery white ventrally. Transition from dorsal to ventral sides marked by alternating indistinct light and dark stripes. Ventral portion of head below center of eye silvery white. Black blotch on upper base of pectoral fin. Spinous dorsal fin membranes brown with dark outer margin; proximal one-fourth of soft dorsal fin brown, distal three-fourths translucent with brown rays; anal fin dark brown with transverse lighter line located three-fourths to the edge of fin. Caudal fin same color and pattern as anal fin. Pelvic fin mostly whitish gray with light brown at base and distal end of fin. Color in alcohol similar to fresh color, except darker brown (Figure 3b).

#### Etymology.

In honor of the late William K Bowes Jr, lead donor of the Hope for Reefs initiative from the California Academy of Sciences, we name *Chromisbowesi* sp. n. A pioneering venture capitalist and visionary Bay Area philanthropist, Bill Bowes was devoted to advancing science and generously supported groundbreaking research spanning across biotech, medical, and other scientific disciplines. The name is a noun in the genitive case.

#### Distribution and habitat.

*Chromisbowesi* sp. n. was found in many localities of the Verde Island Passage, such as in Verde Island, Puerto Galera Bay, and Batangas Bay. The five specimens were recorded over low to moderate complexity habitats between 80 and 120 m depth. Specimens identified as *Chromisearina* were collected by Nishiyama et al. (2012) in Iou-jima Island, Kagoshima Prefecture, Japan, between 70 and 80 m depth, but their mtDNA COI sequence and color description match *Chromisbowesi*, therefore this species is distributed at least to southern Japan. We also sequenced the holotype of *Chromisearina* and its sequence is clearly distinct from *C.bowesi*.

## Discussion

The Verde Island Passage in the Philippines is known as the center of marine biodiversity (Carpenter and Springer 2005). However, little is known about its biodiversity at mesophotic depths (30–150 m) due to the previous lack of technical deep diving (Shepherd et al. 2018). The recent deep diving exploration of this region has led to the discovery of many new species (Anderson et al. 2016, Rocha et al. 2017), including the three species herein presented. Based on the rates of discovery, ongoing exploration should uncover many more species.

Species of *Chromis* are abundant on MCEs (Thresher and Colin 1986, Bejarano et al. 2014, Wagner et al. 2014, Pinheiro et al. 2016), and many of them have been recently discovered and described (e.g., Pyle et al. 2008). Ecological partitioning is known to drive diversification from highly diverse areas to peripheral habitats (Bowen et al. 2013). Thus, we believe that the intense competition on shallow coral reefs could be a factor leading to diversification and colonization of MCEs. The high relative abundance of *Chromis* in MCEs may be related to food (plankton) availability (Pinheiro et al. 2016). While other resources, such as corals and algae, are known to decrease with depth due to a decrease on sunlight, plankton is still abundant on MCEs, sustained mainly by currents, upwelling events and vertical migrations (Thresher and Colin 1986).

Among the new species, coloration and body depth are the most distinctive characters, information commonly used to distinguish *Chromis* species from each other (Allen and Erdmann 2009). Moreover, we compared the COI gene sequence of the new species with more than 60 other *Chromis* species available from GenBank and California Academy of Sciences. The closer relative of *C.gunting* seems to be *C.scotochiloptera*, a shallow water species endemic to the Coral Triangle. The closer relative of *C.hangganan* seems to be *C.pembae*, from shallow waters of the western Indian Ocean, and for *C.bowesi* seems to be *C.earina*, a conspicuous damselfish found in MCEs of the Western and Central Pacific, including the same reefs where we found *C.bowesi*. Although some *Chromis* species are missing for a complete phylogeny and understanding of the evolutionary history of the genus, these results indicate complex speciation processes, which can involve both sympatric and allopatric drivers.

Most of the species described here are only known from MCEs in the Philippines. However, they are not necessarily endemic species, as our knowledge of MCE biodiversity in the Indian and Pacific Oceans is very limited, and deep diving exploration is revealing new records of fishes in many different locations (Wagner et al. 2014, Pinheiro et al. 2015, Simon et al. 2016). For example, *C.earina* and *C.degruyi*, species exclusive to MCEs originally described from central and south Pacific islands (Pyle et al. 2008), are reported here for the first time from the Philippines (CAS 242296, CAS 243185). Moreover, genetic comparisons reveal that the prior report of *C.earina* from Iou-jima Island, Kagoshima Prefecture, Japan (Nishiyama et al. 2012), is actually *C.bowesi*, described in this study. The continued exploration of mesophotic coral ecosystems will likely reveal many more new species, and increase the known ranges of known species.

## Supplementary Material

XML Treatment for Chromis gunting

XML Treatment for Chromis hangganan

XML Treatment for Chromis bowesi
